# Pneumonia Incidence and Mortality in Mainland China: Systematic Review of Chinese and English Literature, 1985–2008

**DOI:** 10.1371/journal.pone.0011721

**Published:** 2010-07-23

**Authors:** Xuhua Guan, Benjamin J. Silk, Wenkai Li, Aaron T. Fleischauer, Xuesen Xing, Xiaoqing Jiang, Hongjie Yu, Sonja J. Olsen, Adam L. Cohen

**Affiliations:** 1 Hubei Center for Disease Control and Prevention, Hubei, People's Republic of China; 2 Respiratory Diseases Branch, Division of Bacterial Diseases, Centers for Disease Control and Prevention, Atlanta, Georgia, United States of America; 3 Division of Emerging Infections and Surveillance Services, Centers for Disease Control and Prevention, Atlanta, Georgia, United States of America; 4 Chinese Center for Disease Control and Prevention, Beijing, People's Republic of China; University of Witwatersrand, South Africa

## Abstract

**Background:**

Pneumonia is a leading infectious disease killer worldwide, yet the burden in China is not well understood as much of the data is published in the non-English literature.

**Methodology/Principal Findings:**

We systematically reviewed the Chinese- and English-language literature for studies with primary data on pneumonia incidence and mortality in mainland China. Between 1985 and 2008, 37 studies met the inclusion criteria. The quality of the studies was highly variable. For children <5 years, incidence ranged from 0.06–0.27 episodes per person-year and mortality ranged from 184–1,223 deaths per 100,000 population. Overall incidence and mortality were stable or decreased over the study period and were higher in rural compared to urban areas.

**Conclusions/Significance:**

Pneumonia continues to be a major public health challenge in young children in China, and estimates of pneumonia incidence and mortality vary widely. Reliable surveillance data and new prevention efforts may be needed to achieve and document additional declines, especially in areas with higher incidence and mortality such as rural settings.

## Introduction

Despite the availability of safe and effective antibiotics and vaccines for treatment and prevention, pneumonia is a leading cause of death worldwide and the leading infectious disease killer [1], [2]. Pneumonia is the single leading cause of death globally among children under 5 years of age accounting for approximately 2 million deaths annually [2], [3]. Children in developing countries have an estimated 0.29 episodes of pneumonia per person-year, compared with 0.05 episodes per person-year in developed countries [3].

Pneumonia is one of the leading causes of death in adults and children in China [4]. In urban areas, pneumonia is the fourth leading cause of death, and in rural areas pneumonia is the leading cause of death [5], [6]. A recent article in the Chinese literature estimated that each year in China there are 2.5 million patients with pneumonia and that 125,000 (5%) of these patients die of pneumonia-related illness [5]. A 2008 global review by Rudan and colleagues estimated that there were 21.1 million new cases of clinical pneumonia annually in China in children under 5 years of age (0.22 episodes/person-year), which is second only to India in burden (43.0 million new cases, 0.37 episodes/person-year) [3]. Available estimates of the burden of childhood pneumonia in China vary widely, and pneumonia accounts for an estimated 17% of all child deaths in China and 67% of all childhood pneumonia deaths in the Western Pacific region [3], [6], [7].

Although the global community recognizes that pneumonia is an important public health issue in China, the disease burden is not well studied nor necessarily reported in the English language and these data have not been systematically reviewed for English- or Chinese-language readers [8]. Complicating an assessment of the pneumonia incidence and mortality in China is the lack of ongoing and systematic surveillance. With the exception of human avian influenza and severe acute respiratory syndrome (SARS), pneumonia and other respiratory diseases are not included in the 39 nationally notifiable infectious diseases in China [9]. However, in the wake of the 2003 SARS outbreak, enhanced surveillance using pneumonia of unknown etiology for early detection of suspected SARS was initiated in 2004 (case definition given in Table S1). We conducted a systematic review of the Chinese- and English-language literature in order to describe pneumonia incidence and mortality in China, evaluate the quality of published studies, and identify gaps in the literature that can be addressed through surveillance and epidemiologic research projects in the future.

## Methods

### Ethics

No ethical review was required since all results are from the published literature.

### Searching

Using PubMed, we searched the National Library of Medicine database for manuscripts published before October 31, 2008. The reference terms “pneumonia,” “acute respiratory infection,” and “lower respiratory tract infection” were each combined with “China” and “Mainland China.” Using equivalent terms, we performed additional searches for publications in the Chinese medical literature using the Wanfang (http://www.wanfangdata.com/) and Chongqingvip (www.cqvip.com) databases [10], [11]. In these databases, publications were available since 1982 and 1989, respectively. The English search terms were translated into Chinese (by author X.G.) for use in the Chinese-language searches [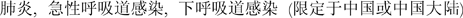]. In addition, journal articles cited in the identified manuscripts were collected and added to the review.

### Selection

**Figure 1:**
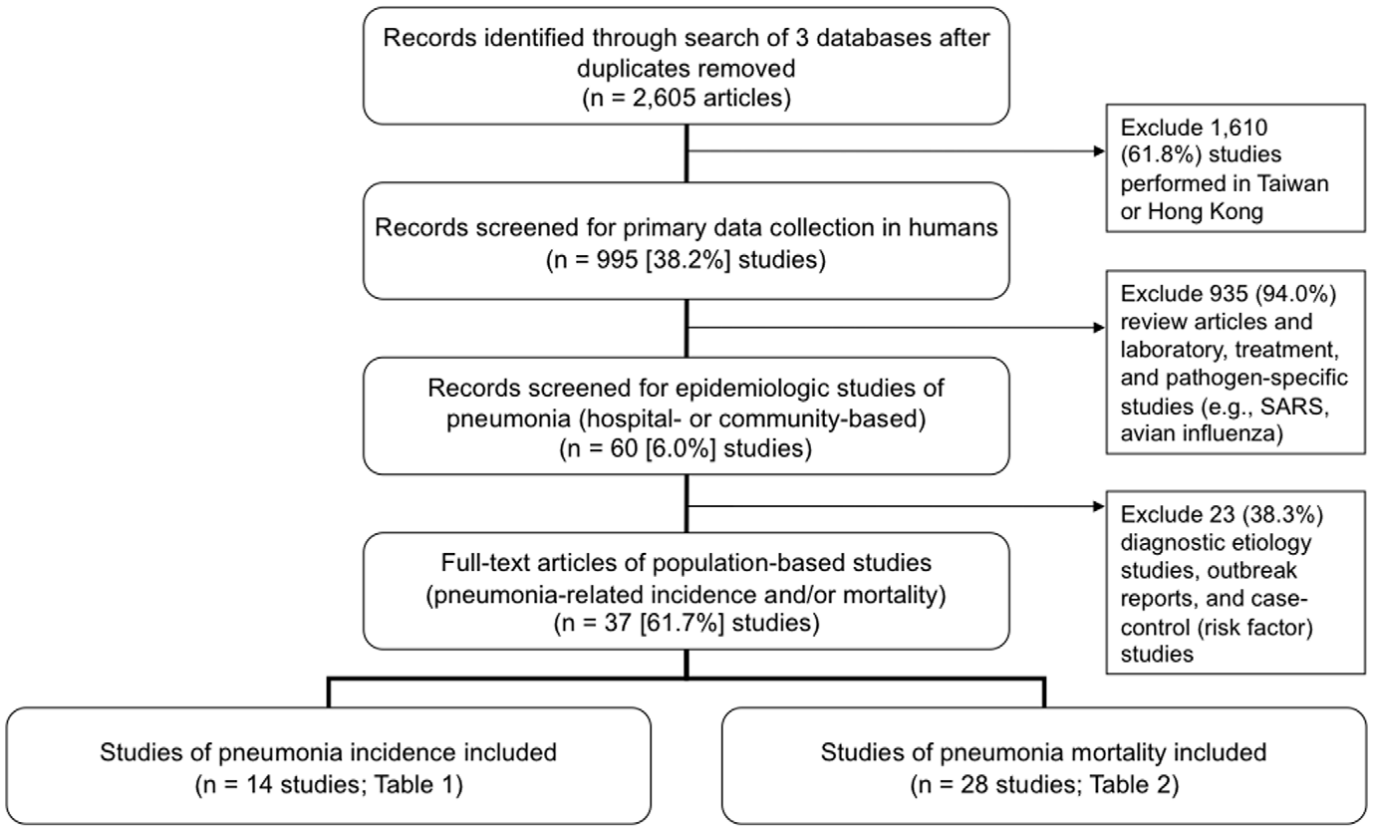
Literature review and inclusion criteria.

Clinical and community-based studies with primary data collection in humans were identified through a literature search conducted in November 2008 (Figure 1). Studies conducted exclusively in Hong Kong special administrative region (SAR), Macao SAR or Taiwan, China, were excluded. We also excluded studies focusing exclusively on SARS and avian influenza, outbreak reports, diagnostic studies of pneumonia etiologies, and studies that did not include population-based estimates of incidence or mortality.

### Validity assessment and data abstraction

Two coauthors who read Chinese as a first language (X.G., W.L.) abstracted from these references the year of publication; province, prefecture, and city; study population (e.g., age group); study design; site of case detection (i.e., inpatient, outpatient, or both); pneumonia case definition; and estimates of pneumonia-related incidence and mortality. English data abstraction was each checked by a native English and Mandarin speaker. Data abstractions were validated during direct discussions with English-speaking epidemiologists (B.J.S., A.T.F., S.J.O., A.L.C.).

### Quantitative data synthesis

**Figure 2:**
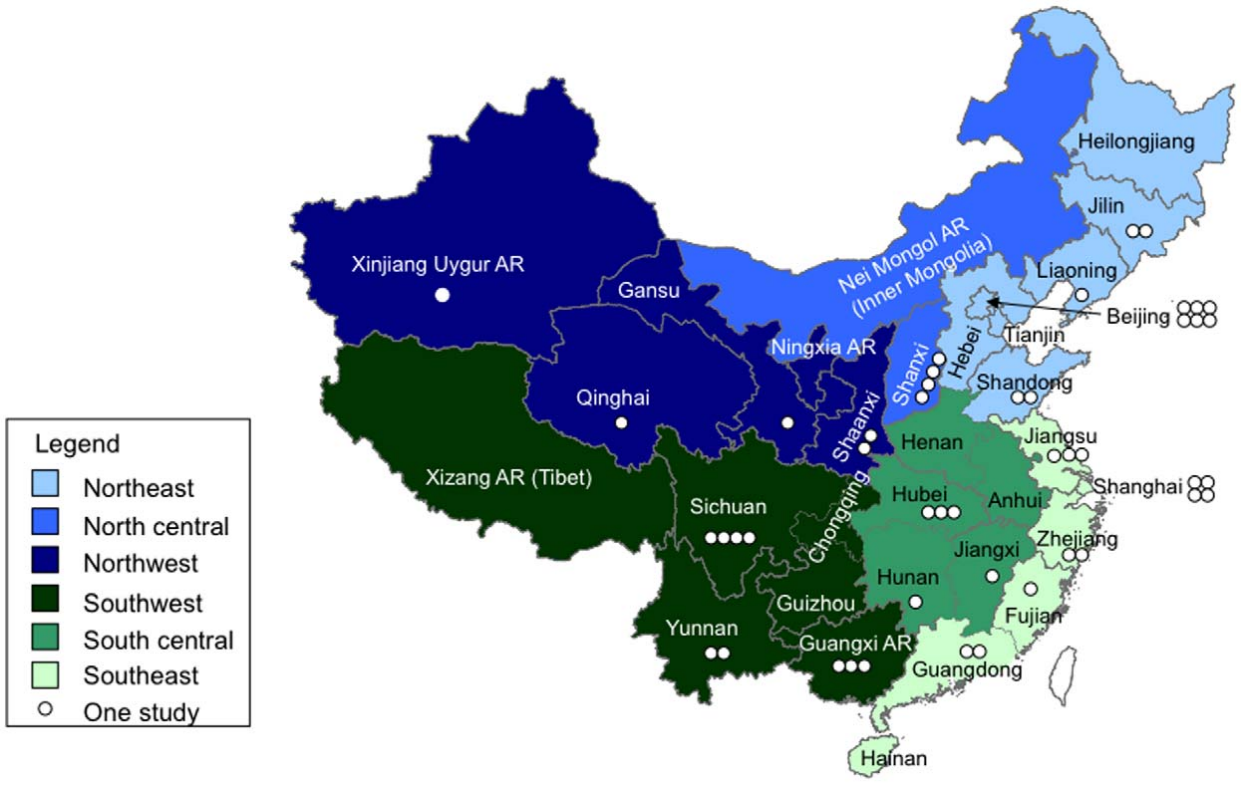
Map of mainland China divided into six regions.

The mainland of China has 31 province-level divisions [22 provinces, 5 autonomous regions (AR), and 4 municipalities (Beijing, Chongqing, Shanghai, Tianjin)]. For the analysis, we divided the country into 6 regions (Figure 2): Northeast (Beijing, Hebei, Heilongjiang, Jinlin, Liaoning, Shandong, Tiangjin,); North Central [Nei Mongol AR (Inner Mongolia), Shanxi]; Northwest (Gansu, Ningxia Hui AR, Shaanxi, Qinghai, Xinjiang Uyghur AR); Southwest [Chongqing, Guangxi AR, Guizhou, Sichuan, Xizang AR (Tibet), Yunnan]; South Central (Anhui, Henan, Hubei, Hunan, Jianxi) and Southeast (Fujian, Guangdong, Hainan, Jiangsu, Shanghai, Zhejiang). Study population types were divided into rural, urban or both according to regulations promulgated by the National Bureau of Statistics of China in 2006 [6].

Incidence is reported as the annual number of pneumonia episodes per year (i.e., person-years). For manuscripts presenting incidence per 100,000 population, data were converted to person-years to be comparable. For example, if there were 200 cases in a year in a population of 10,000, the incidence would be converted from 2,000 per 100,000 per year to 0.02 person-years. When incidence was reported on an annual basis during a multi-year study period within a single study, a simple mean was calculated to report overall incidence during the study period. For mortality measures, when possible, we calculated case fatality rate (i.e., the percentage of pneumonia cases that died), mortality per 100,000 population, and mortality per 1,000 live births annually. We evaluated trends in incidence and mortality and made comparisons across studies by using studies with similar study methods and case definitions when possible.

There were three pneumonia case definitions used in the studies: I. The World Health Organization (WHO) case definition of pneumonia for Integrated Management of Childhood Illness [12], II. Chinese medical association guidelines (IIa: community-acquired pneumonia (CAP) [13] or IIb: hospital-acquired pneumonia (HAP) [14]), and III. physician assessment (IIIa: acute lower respiratory infection; IIIb: newborn pneumonia; or IIIc: pneumonia as a cause of death in children under 5 years of age). The specific signs and symptoms for each case definition are detailed in Table S1.

### Quality evaluation of included studies

For each study that we identified through the systematic review, we critically reviewed the quality of each manuscript. Based on published recommendations for measuring quality of epidemiologic studies of pneumonia [15], we assessed quality using the following six criteria: (1) geographic location was reported, (2) study was conducted for a period of at least one year or multiples of one year to account for seasonal factors, (3) site of case detection or surveillance location was reported, (4) age and population size of cohort of at least 50 cases were reported, (5) quality assurance and monitoring methods were employed to assure that data was complete and high quality, and (6) a clearly defined case definition (e.g., not based solely on clinical diagnosis) was used and reported. These six criteria were selected to represent basic and essential indicators of epidemiologic study quality. Each criteria was dichotomous (1 = reported and 0 = not reported); the sum of all reported criteria yielded the manuscript's overall quality criteria score (0 to 6; Tables S2 and S3). At least one coauthor who read Chinese as a first language (X.G., W.L.) reviewed each of the studies to evaluate these six criteria; evaluations were then validated during direct discussions with an English-speaking epidemiologists (B.J.S., A.T.F., S.J.O., A.L.C.). We included all studies in this review to report the differences in study quality.

## Results

Thirty-seven published studies met the inclusion criteria (Figure 1); 14 publications included data on pneumonia incidence and 28 publications reported pneumonia mortality estimates. At least three studies were conducted in Beijing, Guangxi AR, Hubei, Jiangsu, Shanghai, Shanxi, and Sichuan each (Figure 2). Five (36%) of the 14 incidence articles and 3 (11%) of the 28 mortality articles were identified using Pubmed [16], [17], [18], [19], [20], [21], [22], [23].

### Quality assessment

The studies were each evaluated on six quality indicator criteria (Tables S2 and S3). First, all studies reported the geographic location of the study, but few, if any, reported more specific information on setting such as altitude and annual rainfall, prevalence of malnutrition and AIDS, or immunization coverage against pneumonia vaccines and access to health care [15]. Second, all but 2 studies (35 [95%] of 37) reported at least one year of data. Third, all studies reported the site of case detection and the age of the cases. Fourth, the population size was not given for 15 (54%) of the 28 mortality studies; when population was reported, at least 50 cases were reported. Fifth, four (22%) of 18 studies of incidence and 27 (97%) of 28 studies of mortality reported quality assurance and monitoring.

The sixth quality criteria evaluated the case definition used. Of the 14 studies that reported incidence, 5 (36%) used the WHO case definition for pneumonia and 4 (26%) used the Chinese Medical Association guidelines case definition; 5 (36%) used physician diagnosis, which was considered less reliable than the other case definitions. The incidence estimates using the WHO case definition were generally higher than those using a physician diagnosis, so we report incidence estimates separately by case definition; there was no clear trend in mortality estimates based on case definition, so we did not separate these estimates by case definition. Five (33%) of 15 incidence studies and only 1 (4%) mortality study included chest x-ray in their case definition. Most of the mortality data relied on physician diagnosis (23 [82%] of 28 studies). One quarter (7 of 28) of the mortality studies were reports of deaths for children under 5 years of age collected through the national death surveillance program.

In summary, all of the studies met at least four of the six quality criteria. Only 5 (18%) of the 28 mortality studies and none of the incidence studies met all six quality criteria.

### Incidence

#### Age

Based on 6 studies, the age-specific incidence of pneumonia in children <1 year of age ranged from 0.01–0.68 episodes per person-year using either clinical or WHO case definitions [16], [17], [18], [19], [24], [25]. Based on 9 studies of children <5 years, the incidence ranged from 0.06–0.27 episodes per person-year using clinical case definition and from 0.14–0.66 using the WHO case definition (Table S2) [16], [18], [19], [21], [24], [25], [26], [27], [28]. Incidence was lower in older children [16], [25], and one study presented a pneumonia incidence for adults: 0.037 episodes per person-year for people ≥65 years of age [20].

#### Time trends

A study conducted in two provinces in the West found that pneumonia incidence in children <5 years of age decreased from 1997 to 2000 by 64% in Yunnan province (0.083 in 1997 versus 0.030 episodes per person-year in 2000) and by 47% in Qinghai province (0.017 in 1997 versus 0.009 in 2000) [21].

#### Rural vs. urban areas

Seven incidence studies were performed in urban areas (50%), five in rural areas (36%), and two (14%) in both urban and rural areas. Pneumonia incidence rates were generally higher in rural areas compared with urban areas (Table S2). For example, a study in Guangdong that compared pneumonia rates in both rural and urban settings found that rates in rural areas were more than four times higher than urban rates in children <1 year of age (0.91 vs. 0.19 episodes per person-year, respectively) and more than six times higher in children <5 years of age (0.79 vs. 0.12 episodes per person-year, respectively) [24].

#### Regions

Most (83.3%) studies on pneumonia incidence were conducted in Northeast and Southeast China. The incidence of pneumonia in children <5 years of age in Northeastern China (range: 0.06–0.27 episodes per person-year) [16], [18], [19], [26], was lower than southern China (range: 0.32–0.66 episodes per person-year), which includes the Southeast, South Central, and Southwest regions (Table S2) [24], [27], [28]. There were no studies exclusively in the North Central or Northwest regions. One multi-site study showed that pneumonia incidence among children <5 years of age was higher in Southern than in Northern provinces (0.057 versus 0.013 episodes per person-year in southern Yunnan and northern Qinghai, respectively) [21]. A multi-site study in 1986 reported that pneumonia incidence in children <14 years of age in Eastern China (range 0.041–0.057 episodes per person-year) was higher than in Western and Central China (range 0.032–0.035 episodes per person-year) [25].

### Mortality

#### Age

Twelve (43%) of 28 studies that evaluated pneumonia-related mortality presented estimates for children <1 year of age; 23 (82%) presented estimates for children aged <5 years; most (20 studies, 71%) presented mortality as deaths per 1,000 live births per year (Table S3). Mortality rates ranged from 485 to 890 deaths from pneumonia per 100,000 population for children <1 year of age [25], [29], [30], and from 184 to 1223 deaths from pneumonia per 100,000 population for children <5 years of age [25], [26], [27], [28], [29]. When mortality was measured per 1,000 live births, the estimates ranged from 0.66 to 12.8 deaths for children <1 year of age [23], [31], [32], [33], [34], [35], [36], [37], [38] and from 0.71 to 16 for children <5 years of age [21], [23], [26], [31], [33], [35], [36], [37], [38], [39], [40], [41], [42], [43], [44], [45], [46], [47], [48] (Table S3). Case fatality rates were higher in children <1 year of age (4.67–4.88%) than in children <5 years of age (0.52–1.94%). One study in adults, which included older adults up to age 94 years, found a case fatality rate of 9.98% [22].

#### Time trends

Nine (50%) of 18 studies that examined multiple years of data reported decreasing mortality trends during their study periods [23], [27], [29], [30], [35], [36], [37], [38], [47]. For example, a seven-year study in Shandong province found that the case fatality rate for pneumonia decreased from 1.94% to 0.50% from 1995 to 2001, mortality decreased from 421 to 119 deaths from pneumonia per 100,000 population, and mortality per 1000 live births decreased from 2.50 to 1.07 [26]. However, another nine studies did not show a clear declining trend [21], [33], [40], [41], [42], [43], [44], [45], [46]. For example, a separate study in Shandong province found that while the mortality rate was lowest in the most recent year of the 11-year study (0.14 deaths per 1000 live births in children <5 years in 2006), there was no clear decrease in mortality over the previous decade [31]. No studies showed increasing mortality.

#### Rural vs. urban areas

Nineteen (68%) of 28 mortality studies were conducted in both urban and rural areas, while 6 (21%) were conducted in rural areas and 2 (7%) were conducted in urban areas only. As with incidence, pneumonia mortality in rural areas was generally higher than in urban areas. In one study conducted in Zhejiang from 1991 to 1993, mortality for infants <1 year old ranged from 10.63 to 15.06 cases per 1,000 live births in rural areas compared with 2.29 to 2.54 cases per 1,000 live births in urban areas; for children <5 years, pneumonia mortality ranged from 10.75 to 15.13 in rural areas compared with 2.60 to 2.79 in urban areas [46]. A study in Jiangsu province found 4.18 and 1.12 deaths per 1000 live births in rural and urban areas, respectively [35].

#### Regions

Studies on pneumonia mortality were more geographically representative than studies of pneumonia incidence, and there was at least one study of mortality from each of the six regions (Table S3). Estimates of mortality from pneumonia in infants <1 year old were consistent in four of the six studies conducted in southern China (3.04–3.73 deaths from pneumonia per 1,000 live births) [32], [33], [35], [36]; only one study in northern China reported mortality rates from pneumonia in infants (0.66 deaths from pneumonia per 1,000 live births) [31]. In children aged <5 years, the highest mortality rates were reported by four studies that were each conducted in multiple regions throughout mainland China (9.55–14.40 deaths from pneumonia per 1,000 live births; Table S3) [21], [23], [38], [48]. Relatively high mortality rates in this age group were also reported in the Northwest [42], [43], [44] and the Southwest [37], [47] (3.91–7.51 and 7.7–12.08 deaths from pneumonia per 1,000 live births, respectively).

### Hospital-acquired pneumonia

Three studies evaluated hospital-acquired pneumonia [49], [50], [51]. In Shanghai where the studies were performed, the hospital-acquired pneumonia rate ranged from 1.6% to 2.4% of hospitalized patients.

## Discussion

Given the population size of China (1.3 billion persons) [6], the varied health utilization and economic development, the diverse climate (tropical to subarctic), and the range of population densities, the burden of pneumonia in China would be expected to be large and highly variable across regions. Despite overall trends from these studies suggesting that pneumonia incidence and mortality are stable or decreasing, pneumonia continues to be a major public health concern in China [7]. The studies in this review found incidence of pneumonia in children <5 years of age that were as low as what has been estimated for the developed world globally (0.05 episodes per person-year) and that were as high as what has been estimated for the developing world (0.29 episodes per person-year) [52]. The studies included in this review reported pneumonia incidence for children <5 years of age (0.06–0.27 episodes per person-year from 1985 to 2008) that was similar or less than what has been estimated for China (0.22 episodes per person-year in 2008) [3]. Although the studies reported a wide range of pneumonia mortality estimates (184–1,223 deaths per 100,000 population), these are consistent with pneumonia remaining the leading cause of childhood mortality in China [7]. While the data summarized here provide insights into pneumonia in China, they also serve as a reminder that reliable and high quality national and regional data on pneumonia incidence and etiology are needed to adequately direct prevention and control efforts.

Perhaps the largest limitation to this study is that study comparisons of morbidity and mortality rates were constrained by the wide variation and quality of the study designs. This is particularly evident in the wide range of mortality estimates among the different studies. In general, the incidence estimates were higher when the more standardized WHO case definition was used compared with estimates obtained from physician diagnosis; however, there was significant variability even among studies that used similar case definitions. The use of a standard pneumonia case definition that is designed for surveillance and epidemiologic research would improve generalizability and could allow for direct comparisons of incidence and mortality estimates in China and elsewhere [15], [53]. Most studies spanned multiple years, which would account for differences in seasonality of pneumonia, but a few were conducted for one year or less. Although a few of the studies reported large surveillance populations, many calculated incidence based on relatively small populations or did not report the population under surveillance. Most of these studies were conducted in large, urban centers primarily serving residents of densely-populated areas; few included adults or populations from rural western China. Although many of the studies were conducted prospectively, none calculated incidence using active, population-based surveillance and population denominators were not reliably measured in each study. Until standardized case definitions and appropriate surveillance methodology are applied, pneumonia incidence and mortality estimates should be interpreted cautiously.

In regions for which we identified published data, pneumonia incidence in China appears to be declining and mortality is stable or declining from the 1980s to the 2000s. There are several factors that could have contributed to these changes over time. China experienced substantial economic growth during these years, a trend that was more pronounced in the coastal (Eastern) areas. From the beginning of economic reforms in 1978 to 2006, China's gross domestic product (GDP) increased 5,719% from 362.4 billion RMB to 21,087.1 billion RMB [54]. The income of the average Chinese person also improved during this time period; GDP per capita rose by 4,144% from 379 RMB to 16,084 RMB [54]. Economic development may have led to improvements in healthcare quality and access to health services.

In addition to economic development, China is undergoing dramatic healthcare reform, including government-sponsored healthcare in rural areas [55], [56]. Declines in the incidence of pneumonia are likely attributable to the implementation of pneumonia intervention measures, such as technical training for village doctors, health education to parents, improved pneumonia surveillance and case management, and the use of vaccines against pneumonia in the routine immunization program (namely measles and pertussis). The improved detection and recognition of pneumonias following the SARS, avian influenza and 2009 influenza H1N1 epidemics could lead to more cases of pneumonia being promptly identified and treated. Large scale programs to introduce less polluting cookstoves in China have led to decreases in lung cancer and chronic obstructive pulmonary disease [57], [58]; studies from other countries suggest that reductions in exposure to indoor air pollution from solid fuels used for cooking can also lead to fewer cases of pneumonia [59]. Other strategies, including better access to care, improved hygiene, and better nutrition may need strengthening to effectively reduce the incidence of pneumonia in China [60].

For many Chinese, adequate healthcare remains difficult to access; this review revealed a disparate incidence and mortality of pneumonia across different regions of China [55], [61], some of which is likely due to inequalities in health care. For example, respirators and ventilators for children are not currently available in many county hospitals. Eastern China is more developed, whereas Western China is more rural. According to a report on national maternal and child health endorsed by the China Ministry of Health, UNICEF, and WHO, pneumonia is the leading cause of death in children under 5 years of age in some rural areas, and comprises a larger proportion of deaths in children under 5 years of age as the area becomes more rural [62]. This report also demonstrated the decreasing trends in pneumonia mortality across China, especially in areas where few clinical studies have been completed.

Better access to proven public health interventions, including vaccines, is needed in the public sector in China. Vaccines against *Haemophilus influenzae* type b (Hib), *Streptococcus pneumoniae*, and influenza are not part of routine childhood vaccination programs in many countries worldwide [63], [64]; none of these vaccines are included in the routine childhood immunization schedule in mainland China. However, Hib and influenza vaccines are commonly available in many parts of China through vaccination clinics, and Hong Kong SAR is the first region in China, as well as Asia, where pneumococcal conjugate vaccine will be included in their routine childhood immunization program starting September 2009 [65]. In addition, Hong Kong SAR recommends seasonal influenza vaccine use in high risk groups. Vaccine clinical trials in other countries have estimated that 21% of radiologically confirmed pneumonia is caused by Hib [66] and 36% by pneumococcus [66], [67]; over 10% of hospitalized pneumonia in children in nearby Thailand are due to influenza [68]. Studies within China have suggested that Hib and pneumococcus are common causes of pneumonia in children [69], [70], [71], suggesting that widespread use of these two vaccines, as well as influenza vaccine, could reduce the incidence and mortality of pneumonia in China.

This paper has several strengths, particularly the inclusion of papers published in both the English and Chinese literature. A recent global review on childhood pneumonia incidence included only two of the 14 articles presented here, suggesting that articles not published in English are usually overlooked and difficult to obtain [3], [16], [18]. We did not search three of the five major Chinese-language literature databases (the China National Knowledge Infrastructure China Academic Journals Full-text Database, Chinese Biomedical Literature Database, and Chinese Medical Current Content), so we may not have captured all relevant manuscripts; however, the two databases that we did search include some of the greatest number of journals and articles of the five major databases [10], [11], [72]. In addition, this review includes information on all ages, although only two studies included adults. While global pneumonia prevention efforts often focus on children, the burden of pneumonia in adults and the elderly is also substantial. Importantly, interventions aimed at children may have underappreciated benefits on adults. For example, the introduction of universal childhood pneumococcal vaccination in the United States in 2000 resulted in significant declines in pneumococcal incidence in both children and adults. In 2003, the indirect effect of preventing invasive pneumococcal disease in adults was over twice the direct effect of preventing cases in children [73].

Comprehensive data on pneumonia incidence and mortality are essential for monitoring disease trends, guiding policy decisions, and prioritizing disease prevention and control strategies. For China, accurate information on the incidence and mortality of pneumonia, as well as data on cost, will be central for planning the addition of new vaccines to routine childhood immunization programs. Also, seasonal and pandemic influenza remain ever present global threats. Continued surveillance and consideration of influenza vaccination and other control measures are needed. In collaboration with the U.S. Centers for Disease Control and Prevention (CDC), China's CDC is implementing active, population-based surveillance for pneumonia in certain areas using a standard approach and case definitions. The surveillance system will aim to better define pneumonia incidence, identify etiologies, and guide important clinical and public health decisions. Increased laboratory capacity needs to be built to ensure continued rigorous surveillance and quick response to emerging threats. Together these improved surveillance and laboratory data should help improve detection, prevention and control of pneumonia throughout China.

## Supporting Information

Table S1 Pneumonia case definitions used in the studies reviewed.
(0.06 MB DOC)

Table S2 Pneumonia incidence in China, by region.
(0.10 MB DOC)

Table S3 Pneumonia mortality in China, by region.
(0.18 MB DOC)
