# Supplementary material for: Pneumonia Incidence and Mortality in Mainland China: Systematic Review of Chinese and English Literature, 1985–2008
Source: PLoS One. 2010 Jul 23;5(7):e11721. doi: 10.1371/journal.pone.0011721 (PMC2909231; doi:10.1371/journal.pone.0011721)
Supplement: Table S1 — Pneumonia case definitions used in the studies reviewed. (0.06 MB DOC) [file pone.0011721.s001.doc]

Table 1. Pneumonia case definitions used in the studies reviewed

| No. | Source | Signs and Symptoms |
| --- | --- | --- |
| I | WHO Integrated Management of Childhood Illness pneumonia case definition [74] | A case of pneumonia was defined as a patient with cough or difficulty breathing **and** breathing >50/minute for infant aged 2 months to <1 year or >40/minute for child aged 1 to 5 years **and** no chest indrawing, stridor or danger signs. Severe pneumonia: Pneumonia as defined above **and** any general danger sign or chest indrawing or stridor in a calm child. General danger signs for children aged 2 months to 5 years: Unable to drink or breast feed, vomits everything, convulsions, lethargic or unconscious |
| II | Chinese Medical Association guidelines |  |
|  | a. Community-acquired pneumonia | A suspect case pneumonia was defined as a patient with chest radiograph with lobar or segmental consolidation, interstitial change, with or without pleural effusion AND ≥1of the following four signs, symptoms, or laboratory tests:  1) Recent cough, sticky sputum, or aggravating existent respiratory symptoms and purulent sputum, with or without pleural pain **or**  2) Fever **or**  3) Consolidation from physical examination and/or moist rales **or**  4) WBC>10x10^9^/L or WBC<4x10^9^/L, increase in WBC early stage  Patients are excluded if they have tuberculosis, pulmonary tumor, noninfectious interstitial pulmonary disease, edema, atelectasis, embolism, pulmonary infiltrate with eosinophilia syndrome or pulmonary vasculitis. |
|  | b. Hospital-acquired pneumonia | A suspect case of pneumonia was defined as a patient with >2 of the following symptoms that occurred at least 48 hours after hospital admission in a patient admitted without pneumonia:  1) Cough, sputum or aggravating existent symptoms with purulent sputum, with or without chest ache;  2) Fever;  3) Moist rales or consolidation on physical examination;  4) Recent atypical symptoms (lethargy, unconsciousness, limited activity, decreased balance, incontinence, anorexia, malaise, nausea, vomiting, diarrhea, hyperpnoea) in a patient who cannot get a chest radiograph because they are bed ridden or have a disability. |
| III | Physician assessment |  |
|  | a. Pneumonia | Lower respiratory infections were defined as bronchitis, asthmatic bronchitis, bronchiolitis or pneumonia. Acute respiratory infection includes upper respiratory infections defined as cold, pharyngitis, laryngitis, tonsillitis, or tympanitis. |
|  | b. Newborn pneumonia | Includes aspirated pneumonia and infectious pneumonia |
|  | c. Pneumonia death in children <5 years | Pneumonia as cause of death according to the International Classification of Diseases and Related Health Problems (ICD, version 9 or 10) |
| IV | Pneumonia of unknown etiology (nationally notifiable disease) | A case of pneumonia was defined as a patient with all of the following criteria without specific laboratory diagnosis:   1. Fever (temperature >38℃) 2. Radiological evidence of pneumonia or acute respiratory distress syndrome (ARDS) 3. Normal white blood cell count (WBC; range 4–10×109 cells per L), leukopenia (WBC <4×10^9^ cells per L), or lymphopenia (lymphocyte count <0.8×10^9^ cells per L) at clinical presentation 4. Absence of clinical improvement after 3-5 days treatment with broad-spectrum antibiotics |
