# Supplementary material for: Pneumonia Incidence and Mortality in Mainland China: Systematic Review of Chinese and English Literature, 1985–2008
Source: PLoS One. 2010 Jul 23;5(7):e11721. doi: 10.1371/journal.pone.0011721 (PMC2909231; doi:10.1371/journal.pone.0011721)
Supplement: Table S2 — Pneumonia incidence in China, by region. (0.10 MB DOC) [file pone.0011721.s002.doc]

Table S2. Pneumonia incidence in China, by region

| Reference (author name and publication year) | Study period* | Province* | Urban/  rural | Study design | Site of case detection*† | Age | Population size* | Case definition*‡ | Quality assurance and monitoring* | Quality criteria score (out of 6*) | Incidence (in person-years, unless otherwise specified) | | |
| --- | --- | --- | --- | --- | --- | --- | --- | --- | --- | --- | --- | --- | --- |
|  |  |  |  |  |  |  |  |  |  |  | Children <1 year of age | Children <5 years of age | Adults or all ages |
| **Northeast** |  |  |  |  |  |  |  |  |  |  |  |  |  |
| Zhang (1986)[16] | 2 years  (7/1981-6/1983) | Beijing | Rural | Prospective | Both | <12 years | 526 persons | IIIa | Yes | 5 | 0.079 | 0.069 | 0.026 |
| Li (1985)[17] | 1 year  (1/1982-12/1982) | Beijing | Both | Prospective | Both | 0-28 days | 19,571 live births | IIIb, XR | No | 4 | 0.011-0.013 | NA | NA |
| Zeng (1987)[75] | 1 year  (9/1984-8/1985) | Beijing | Urban | Prospective | Both | 6 months-  7 years | 1,312 persons | IIIa | No | 4 | 0.24 | 0.059 | 0.0066 |
| Zhang (1990)[19] | 3 years  (9/1984-8/1987) | Beijing | Urban | Prospective | Both | 6 months-  7 years | 1,063 person-years | IIIa | Yes | 5 | 0.68 | 0.27 | 0.027 (<7 years) |
| Gao (2004)[26] | 7 years  (01/1995-12/2001) | Shandong | Urban | Prospective | Both | <5 years | 321,249 persons | I | No | 5 | NA | 0.22 | NA |
|  |  |  |  |  |  |  |  |  |  |  |  |  |  |
| **Southeast** |  |  |  |  |  |  |  |  |  |  |  |  |  |
| Zhou (2000)[24] | 5 years  (10/1993-9/1998) | Guangdong | Both | Prospective | Both | <5 years | 120,970 persons | I | No | 5 | 0.46 | 0.32 | NA |
| Xie (2003)[49] | 5 years  (7/1998-6/2003) | Shanghai | Urban | Prospective | Inpatient | Range not given | 118,215 inpatients | IIb, XR | No | 5 | NA | NA | 2.4% |
| Hu (2002)[50] | 1 month  (10/1999) | Shanghai | Urban | Retrospective | Inpatient | Range not given | 19,235 inpatients | IIb, XR | Yes | 5 | NA | NA | 2.1% |
| Deng (2003)[51] | 3 years  (1/2000-12/2002) | Shanghai | Urban | Retrospective | Inpatient | Range not given | 2,891,859 persons | IIb, XR | No | 5 | NA | NA | 1.6% |
| Bai (2007)[20] | 1 month  (2/2004-3/2004) | Shanghai | Urban | Prospective | Both | ≥ 65 years | 16,000 persons | IIa, XR, C/R+ | No | 4 | NA | NA | 0.037  (adults ≥65 years) |
| **South Central** |  |  |  |  |  |  |  |  |  |  |  |  |  |
| Xie (2003)[27] | 3 years  (01/1997-12/2000) | Hubei | Rural | Prospective | Both | <5 years | 75,376 persons (total) | I | No | 5 | NA | 0.64 | NA |
| **Southwest** |  |  |  |  |  |  |  |  |  |  |  |  |  |
| Xu (2000)[28] | 3 years  (1/1995-12/1997) | Yunnan | Rural | Prospective | Both | <5 years | 6,966 persons (total) | I | No | 5 | NA | 0.66 | NA |
| **Multiple regions** |  |  |  |  |  |  |  |  |  |  |  |  |  |
| Lin (1990)[25] | 1 year  (1/1986-12/1986) | 6 sites (Hubei, Shanxi, Guangxi, Jiangsu, Sichuan, Jilin) | Rural | Prospective | Both | 0-14 years | 90,068 persons | IIIa | Yes | 5 | 0.16 (range 0.13-0.19) | 0.094 | 0-14 years  0.035 (range 0.22-0.057) |
| Wang (2003)[21] | 4 years  (1/1997-12/2000) | 2 sites (Yunnan,  Qinghai) | Rural | Retrospective | Both | <5 years | 9,516 live births | I | No | 5 | NA | 0.14 | NA |

NA = Not applicable, XR = X-ray performed as part of case definition

* The six criteria used to evaluate the quality of each study were based on the six variables marked with an asterisk: (1) geographic location reported, (2) study period of at least one year or multiples of one year to account for seasonal factors, (3) site of case detection or surveillance location reported, (4) age and population size of cohort reported, and at least 50 cases reported, (5) clearly defined case definition (e.g., not based solely on clinical diagnosis), and (6) quality assurance and monitoring methods employed to assure that data was complete and of high quality.

† Inpatient, outpatient, or both inpatient and outpatient

‡ I. The World Health Organization (WHO) case definition for Integrated Management of Childhood Illness, II. Chinese medical association guidelines (IIa: community-acquired pneumonia (CAP) or IIb: hospital-acquired pneumonia (HAP)), and III. physician assessment (IIIa: acute lower respiratory infection; IIIb: newborn pneumonia; or IIIc: pneumonia as a cause of death in children under 5 years of age) Please refer to Table 1 for full case definitions.
