# Supplementary material for: Pneumonia Incidence and Mortality in Mainland China: Systematic Review of Chinese and English Literature, 1985–2008
Source: PLoS One. 2010 Jul 23;5(7):e11721. doi: 10.1371/journal.pone.0011721 (PMC2909231; doi:10.1371/journal.pone.0011721)
Supplement: Table S3 — Pneumonia mortality in China, by region. (0.18 MB DOC) [file pone.0011721.s003.doc]

Table S3. Pneumonia mortality in China, by region

| Reference (author name and publication year) | Study period* | Province* | Urban/  rural | Study design | Site of case detection*† | Age | Population size | Case definition*‡ | Quality assurance and monitoring | Quality criteria score (out of 6*) | Mortality measures  (A. Case-fatality %, B. Mortality per 100,000 population, C. Mortality per 1,000 live births) | | |
| --- | --- | --- | --- | --- | --- | --- | --- | --- | --- | --- | --- | --- | --- |
|  |  |  |  |  |  |  |  |  |  |  | Children <1 year of age | Children <5 years of age | Adults or all ages |
| **Northeast** |  |  |  |  |  |  |  |  |  |  |  |  |  |
| Gao (2004)[26] | 7 years  (1/1995-12/2001) | Shandong | Urban | Prospective | Both | <5 years | 321,249 | IIIa | Yes | 5 | NA | A. 1.09%  B. 239  C. 1.93 | NA |
| Wang (2008)[39] | 5 years  (1/2001-12/2005) | Liaoning | Both | Prospective | Both | <5 years | Not given | IIIc | Yes | 4 | NA | C. 0.85 | NA |
| Xue (2006)[22] | 2 years  (7/2002-6/2004) | Beijing | Urban | Prospective | Both | 18-94 years | 451 | IIa, XR | Yes | 6 | NA | NA | A. 9.98% |
| Deng (2008)[31] | 11 years  (1/1996-12/2006) | Shandong | Both | Prospective | Both | <5 years | Not given | IIIc | Yes | 4 | C. 0.66 | C. 0.72 | NA |
|  |  |  |  |  |  |  |  |  |  |  |  |  |  |
| **North Central** |  |  |  |  |  |  |  |  |  |  |  |  |  |
| Xiao (2008)[40] | 5 years  (1/2001-12/2005) | Shanxi | Both | Prospective | Both | <5 years | Not given | IIIc | Yes | 4 | NA | C. 3.00 | NA |
| Yan (2008)[41] | 6 years  (1/2001-12/2006) | Shanxi | Both | Prospective | Both | <5 years | Not given | IIIc | Yes | 4 | NA | C. 0.71 | NA |
|  |  |  |  |  |  |  |  |  |  |  |  |  |  |
| **Northwest** |  |  |  |  |  |  |  |  |  |  |  |  |  |
| Jiang (2005)[76] | 20 years  (1/1978-12/1997) | Gansu | Both | Retrospective | Inpatient | <28 days | 436 | IIIb | No | 4 | NA | NA | A. ≤28 days: 18.6% |
| Ye (2008)[42] | 9 years  (1/1997-12/2005) | Shaanxi | Both | Prospective | Both | <5 years | Not given | IIIc | Yes | 4 | NA | C. 6.03 | NA |
| Hu (2008)[43] | 6 years  (1/2000-12/2005) | Shaanxi | Both | Prospective | Both | <5 years | Not given | IIIc | Yes | 4 | NA | C. 7.51 | NA |
| Liu (2008)[44] | 5 years  (1/2001-12/2005) | Xinjiang | Both | Prospective | Both | <5 years | Not given | IIIc | Yes | 4 | NA | C. 3.91 | NA |
|  |  |  |  |  |  |  |  |  |  |  |  |  |  |
| **Southeast** |  |  |  |  |  |  |  |  |  |  |  |  |  |
| Li (2001)[32] | 1 year  (1991) | Jiangsu | Both | Prospective | Death case report | 0-5 years | Not given | IIIc | Yes | 4 | C. 3.73 | NA | C. 1-4 years: 0.05 |
| Chen (2000)[33] | 5 years  (1/1995-12/1999) | Fujian | Both | Prospective | Death case report | <5 years | 1,638,000 | IIIc | Yes | 5 | C. 3.51 | C. 5.68 | C. ≤28 days: 2.31 |
| Jiang (2002)[34] | 1 year  (2000) | Guangdong | Both | Prospective | Death case report | <5years | Not given | IIIc | Yes | 4 | C. 1.84 | NA | C. 1-4 years 0.202 |
| Shen (2008)[45] | 10 years  (1/1997-12/2006) | Zhejiang | Both | Prospective | Both | <5 years | Not given | IIIc | Yes | 4 | NA | C. 0.89 | NA |
| Lan (2008)[46] | 11 years  (1/1997-12/2007) | Zhejiang | Both | Prospective | Both | <5 years | Not given | IIIc | Yes | 4 | NA | C. 2.38 | NA |
|  |  |  |  |  |  |  |  |  |  |  |  |  |  |
| **South Central** |  |  |  |  |  |  |  |  |  |  |  |  |  |
| Xie (2003)[27] | 3 years  (6/1997-2/2000) | Hubei | Rural | Prospective | Both | <5 years | 75,376 (total) | I | Yes | 6 | NA | A. 0.52%  B. 327 | NA |
| Yang (2008)[35] | 10 years  (1/1998-12/2007) | Hunan | Both | Prospective | Both | <5 years | Not given | IIIc | Yes | 4 | C. 3.33 | C. 3.62 | NA |
| Luo (2008)[36] | 5 years  (1/2002-12/2006) | Jiangxi | Not given | Prospective | Both | <5 years | Not given | IIIc | Yes | 4 | C. 3.04 | C. 2.30 | NA |
|  |  |  |  |  |  |  |  |  |  |  |  |  |  |
| **Southwest** |  |  |  |  |  |  |  |  |  |  |  |  |  |
| Xu (2000)[28] | 3 years  (1/1995-12/1997) | Yunnan | Rural | Prospective | Both | <5 years | 2150-2460 (depending on year) | I | Yes | 6 | NA | A. 0.57%  B. 359 | NA |
| Liang (2003)[29] | 7 years  (1/1995-12/2001) | Guangxi | Rural | Prospective | Death case report | <5 years | 188,400 | IIIc | Yes | 5 | B. 890 | B. 1223 | B. ≤28 days: 394  1-4 years: 338 |
| Yang (2008)[47] | 5 years  (1/2001-12/2005) | Sichuan | Both | Prospective | Both | <5 years | Not given | IIIc | Yes | 4 | NA | C. 12.08 | NA |
| Huang (2008)[37] | 2 years  (1/2005-12/2006) | Sichuan | Both | Prospective | Both | <5 years | Not given | IIIc | Yes | 4 | C. 5.1 | C. 7.7 | NA |
|  |  |  |  |  |  |  |  |  |  |  |  |  |  |
| **Multiple regions** |  |  |  |  |  |  |  |  |  |  |  |  |  |
| Lin (1990)[25] | 1 year  (1/1986-12/1986) | Hubei, Shanxi, Guangxi, Jiangsu, Sichuan, Jilin | Rural | Prospective | Both | 0-14years | 90,068 | IIIa | Yes | 5 | A. 4.67%  B. 740 | A. 1.94%  B. 184 | A. 0-14 years: 1.76%;  5-14 years: 0.3%  B. 0-14 years: 63.31  5-14 years: 1.80 |
| Shimouchi (1995)[23] | 5 years  (1/1986-12/1990) | Beijing (Nanbu, Shifang, and Shunyian) | Both | Prospective | Both | <5 years | 2,147,000 (total) | I | Yes | 6 | C. 12.8 | C. 14.4 | NA |
| Liu (1990)[30] | 2 years  (1/1987-12/1988) | Hubei, Shanxi, Guangxi, Jiangsu, Sichuan, Jilin | Rural | Prospective | Both | <15 years | 90,068 | I | Yes | 6 | A. 4.88%¶  B. 485 | NA | A. <15 years: 3.29%¶  B. <15years: 44.67* |
| Liu (1996)[38] | 3 years  (1/1991-12/993) | 81 counties or cities in all 30 provinces at the time | Both | Prospective | Death case report | <5 years | 8,550,000 (total) | IIIc | Yes | 5 | C. 11.08 | C. 13.19 | C. 1-4 years: 0.42 |
| Wang (2003)[21] | 7 years  (1/1995-12/2001) | Yunnan, Qinghai | Rural | Retrospective | Death case report | <5 years | 9516 (total live births) | IIIc | Yes | 5 | NA | C. 16 | NA |
| Wang (2005)[48] | 5 years  (1/1996-12/2000) | 116 counties in China in all 31 provinces | Both | Prospective | Death case report | <5 years | 13,000,000 (total) | IIIc | Yes | 5 | NA | C. 9.55 | NA |

NA = not applicable, XR = X-ray performed as part of case definition

* The six criteria used to evaluate the quality of each study were based on the six variables marked with an asterisk: (1) geographic location reported, (2) study period of at least one year or multiples of one year to account for seasonal factors, (3) site of case detection or surveillance location reported, (4) age and population size of cohort reported, and at least 50 cases reported, (5) clearly defined case definition (e.g., not based solely on clinical diagnosis), and (6) quality assurance and monitoring methods employed to assure that data was complete and of high quality.

† Inpatient, outpatient, both inpatient and outpatient, or death case reports=

‡ I. The World Health Organization (WHO) case definition for Integrated Management of Childhood Illness, II. Chinese medical association guidelines (IIa: community-acquired pneumonia (CAP) or IIb: hospital-acquired pneumonia (HAP)), and III. physician assessment (IIIa: acute lower respiratory infection; IIIb: newborn pneumonia; or IIIc: pneumonia as a cause of death in children under 5 years of age) Please refer to Table 1 for full case definitions.

¶ With standard management of acute respiratory infection
